# Digital Evaluation of Vertical Ridge Augmentation with the Modified Shell Technique Using a Xenogeneic Bone Lamina: A Case Series

**DOI:** 10.3390/jcm12227013

**Published:** 2023-11-09

**Authors:** Arndt Happe, Sarah M. Blender, Ralph G. Luthardt, Heike Rudolph, Katharina Kuhn

**Affiliations:** Department of Prosthetic Dentistry, Center of Dentistry University of Ulm, 89081 Ulm, Germany; sarah.blender@uniklinik-ulm.de (S.M.B.); ralph.luthardt@uniklinik-ulm.de (R.G.L.); heike.rudolph@uniklinik-ulm.de (H.R.); katharina.kuhn@uniklinik-ulm.de (K.K.)

**Keywords:** CBCT, 3D evaluation, Boolean operation, vertical bone augmentation, modified shell technique, bone lamina

## Abstract

Vertical ridge augmentation is a demanding and technique-sensitive surgical procedure. In the present case series, cone beam CT (CBCT) scans from the clinical routine of patients treated using a novel approach for vertical bone augmentation were assessed. All patients showed a single-tooth class 5 defect and were treated using a modification of the original shell technique. Cortical bone plates were replaced with a lamina composed of a partially demineralized porcine xenograft. CBCT scans of six consecutive patients were treated with the lamina and particulate bone from the mandibular ramus prior to a single tooth implant in the anterior maxilla were included. Pre- and postsurgical CBCT data sets were superimposed and analyzed digitally using surface matching and Boolean subtraction. The volume of the grafted area was calculated with and without the xenograft. The vertical gain of the ridge height measured in this case series varied from 7 to 11.3 mm. The mean vertical gain was 8.97 mm. The mean volume including the xenograft was 382.59 mm^3^ (SD 73.39) and 250.84 mm^3^ (SD 53.67) without the lamina. The modified shell technique used in this case series for the vertical augmentation of single-tooth class 5 defects provided sufficient bone for single implant restorations.

## 1. Introduction

Vertical ridge augmentation is a demanding and technique-sensitive surgical procedure [1,2]. As a result of the limited number of bony walls in vertical defects in these clinical situations, stabilization of the graft is challenging and it is biologically difficult for angiogenesis because the vessels have to travel a longer distance from the local bone through the graft for bone formation [3,4]. In addition, soft tissue management is demanding. The larger the augmented volume, the more soft tissue has to be mobilized and advanced to cover the area; key elements for success are appropriate graft stabilization and appropriate soft-tissue management [5].

Benic and Hämmerle performed a literature review and introduced a classification for bone defects and linked different defect morphologies to recommendations for treatment options [6]. The classification describes clinical situations of localized alveolar ridge defects with an increasing complexity, from class 0 (optimal contour) to class 5 (vertical defect). They concluded that demanding bone defects like severe horizontal and vertical ridge defects require a staged approach, where autogenous bone is used to augment the site prior to implant placement. There is a consensus that the selection of the optimal technique for the management of vertical ridge augmentation depends on various factors, including the magnitude of the defect, the grafted bone substitute material available, the medical status of patient, and the surgeon’s skills and experience [7]. 

Experimental histo-morphometric analysis has shown that bone blocks from the mandibular ramus used as onlay bone grafts show a high amount of over 50% necrotic bone and only 27.5 % vital bone, even after 6 months of healing [8]. An experimental histological comparison of the sites that were augmented with autogenous bone blocks and particulate autogenous bone grafts in conjunction with GBR in the same patient revealed a faster bone turn over, higher degree of bone remodeling, and higher percentage of vital bone in the specimens of the sites augmented with particulate bone grafts [9]. That is why some authors prefer and advocate for the use of particulate bone grafts over bone blocks today [9,10,11]. 

Khoury introduced a method for grafting these particular demanding three-dimensional ridge defects in 2007 [12]. This technique, called the “split bone block technique” (SSB) or “shell technique”, involves thin cortical plates harvested from the ramus in order to reconstruct the cortical walls. The resulting space is filled with particulate bone harvested from the same site [10,12]. 

In the present case series, a modification of the original SBB technique was evaluated three-dimensionally using data sets of cone beam computer tomography that were taken during clinical routine. The cortical bone plates used to build the bony housing for regeneration were replaced using a partially demineralized porcine xenograft (bone lamina hard, Osteobiol Tecnoss, Torino, Italy). This porcine xenograft has been used for many years in maxillofacial surgery for various indications and has been proven to be clinically successful [13,14,15].

Cone beam computed tomography (CBCT) has become routine in digital radiographic diagnostics and is recommended as routine prior to treatment involving dental implants and bone augmentation procedures [16]. In a staged approach, CBCT scans are used prior to bone augmentation to evaluate the anatomy of the defect, including the adjacent anatomical structures and after healing of the site, in order to plan for the ideal implant position [17,18]. Hence, CBCT scans routinely taken during treatment provide an option to evaluate the amount of bone that has been augmented. Regarding linear measurement, CBCT scans have demonstrated a high degree of reliability and reproducibility for these measurements [16] but, this refers to measurements within one data set.

Usually, if two data sets of CBCT data have to be compared, reference points have to be determined in both data sets in order to calibrate measurements, and considerable errors might occur using this method. Uploading the data to one software platform in order to align the data and compare them facilitates the evaluation of such data. 

This case series article demonstrates a digital technique where two data sets of CBCT scans are superimposed on a software platform in order to evaluate and measure the amount of augmented bone after augmentation with the modified shell technique. The demonstrated technique is widely used in other areas of medical digital imaging and is called a “Boolean operation”. Boolean operations in computer-aided design or computer graphics are a set of operations (e.g., intersection, union, and subtraction) between two objects (e.g., a patient model and an implant model) that are important for performing accurate and reproducible virtual surgical planning [18].

The aim of this case series is to three-dimensionally evaluate a novel approach for vertical bone augmentation with a modified SBB technique using digitally superimposed data sets of CBCT scans. 

## 2. Materials and Methods

The CBCT scans of 6 consecutive patients (3 male and 3 female) who were treated between January and December 2019 with vertical bone augmentation prior to a single tooth implant in the anterior maxilla were included in this case series. Patients’ age ranged from 27 to 55 years, with a mean of 37.5. All patients during that period who met the inclusion criteria were included in the study. Patients were treated in a dental office by an oral surgeon who specialized in bone augmentation and dental implants (AH).

Inclusion criteria were as follows:

Healthy patient, according to ASA class 1 [19]

Smoking less than 5 cigarettes per day

Single tooth gap in the anterior maxilla, at least 6 weeks after extraction

Class 5 defect [6] and the absence of palatal bone wall

Bone augmentation procedure with hard lamina and particulated autogenous bone

Existing high quality CBCT scan prior to augmentation and second CBCT at least 4 months with a maximum of 5 months after surgery

Implant and implant restoration in place for at least 1 year without complications

Informed written consent

### 2.1. Surgical Technique

**Figure 1:**
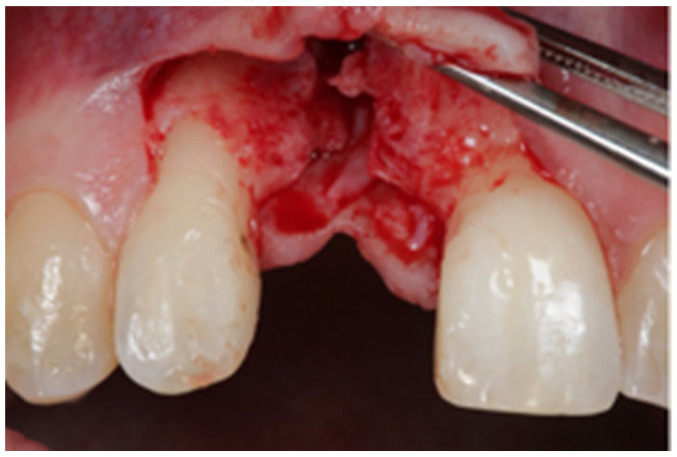
Localized vertical ridge defect. No palatal wall present.

**Figure 2:**
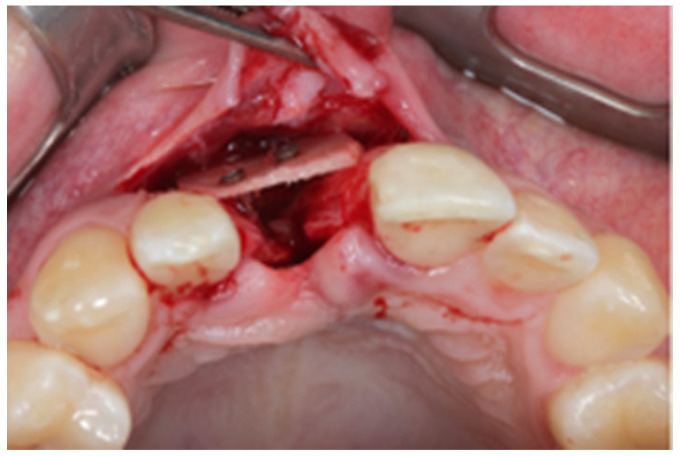
Reconstruction of the buccal wall with porcine lamina.

**Figure 3:**
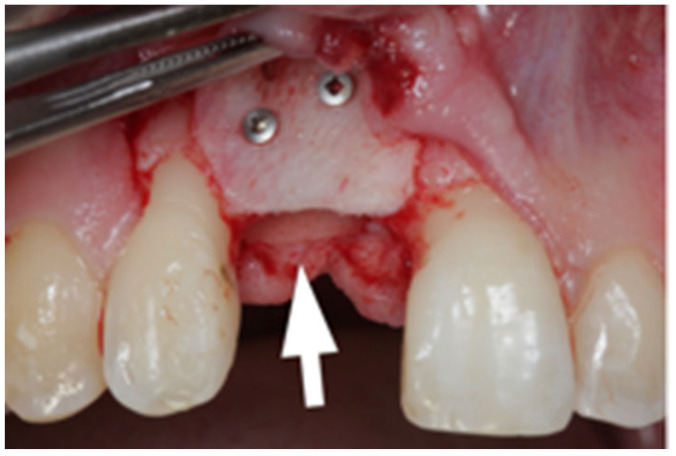
Reconstruction of the buccal and palatal wall (arrow shows palatal wall).

**Figure 4:**
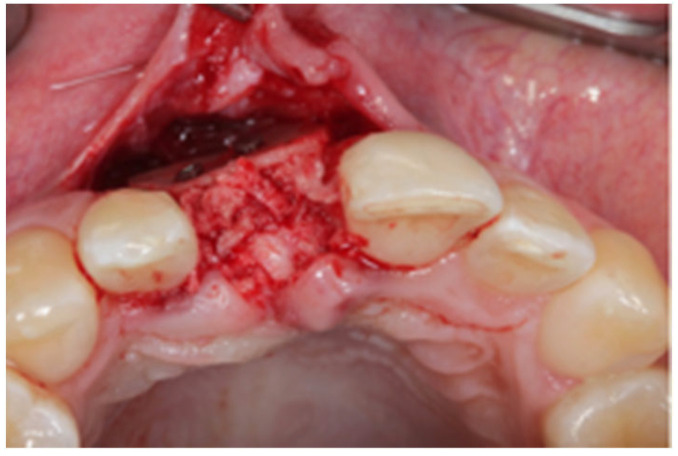
Space filled with autogenous bone grafts.

**Figure 5:**
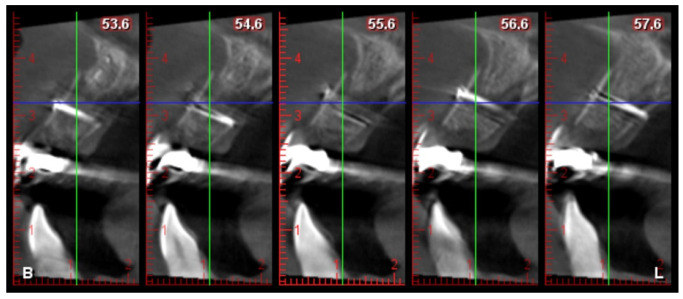
Post-operative CBCT cross sections of the augmented site.

**Figure 6:**
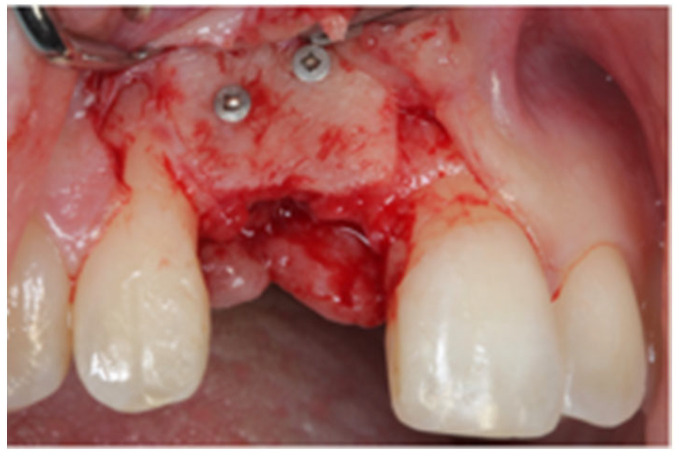
Site 4 months after augmentation. The lamina shows good integration.

**Figure 7:**
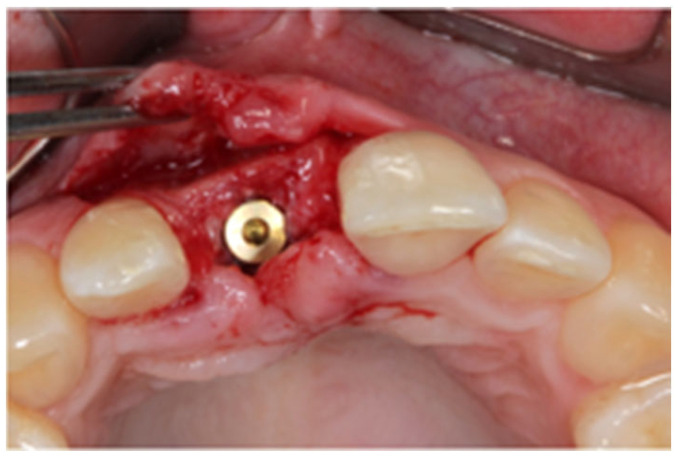
The implant (diameter 3.8 mm) was placed in autogenous bone.

After written consent and preoperative clinical and radiographic diagnosis (CBCT scans with 0.2 mm voxel size resolution), all six patients received 500 mg Amoxicillin or 300 mg Clindamycin as preoperative medication. The surgeries were performed under local anesthesia using Ultracain DS-forte 4% with 1:100,000 Adrenalin (Septodont, Germany). A mucoperiosteal flap was lifted and all remaining soft tissue was cleaned out of the defect (Figure 1). After careful rehydration in a sterile saline solution for at least 10 min, a rigid lamina of 0.7 mm thickness (Osteobiol porcine rigid lamina, Tecnoss, Italy) was tailored to fit the defect in order to reconstruct the buccal and palatal wall of the ridge. The laminas were fixated with osteosynthesis screws (KLS-Micro Module, 1 mm osteosyntheses screws, Carl Martin, Germany) in a way that they rebuild the buccal and palatal cortical plate and created space for augmentation (Figure 2 and Figure 3). Autogenous bone was taken from the mandibular ramus with a trephine, particulated with a bone mill (Quetin, Germany), and placed in between the laminas (Figure 4). The site was covered with a collagen membrane (Osteobiol Evolution, Tecnoss, Italy) and meticulous micro-surgical soft tissue closure was performed. After at least 4 months of healing, the sites were reevaluated using CBCT scans in order to plan for implant placement (Figure 5). At the time of implant placement, the osteosynthesis screws were removed and the implants were placed and covered for submerged healing (Figure 6 and Figure 7). After another 3 months of healing, the implants were uncovered in a second staged surgery and clinically and radiographically (digital periapical x-ray) checked for osseointegration. Afterwards, the treatment was finalized with the restorative treatment. 

### 2.2. D-Evaluation Procedure

All CBCT scans were created using one cone beam computer tomography system (ProMax 3D Classic, Planmeca, Finnland). DICOM data sets were exported in order to analyse the 3D images. 

To evaluate the augmented bone volume with respect to the pre-surgery situation, the pre-surgery and post-surgery volumes were compared.

Data were obtained as follows:

### 2.3. Cone Beam CT Conversion to STL by Means of Segmentation

**Figure 8:**
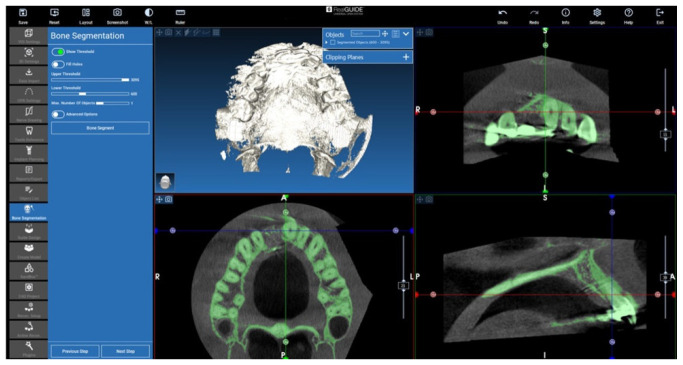
Threshold automatic pixel selection.

**Figure 9:**
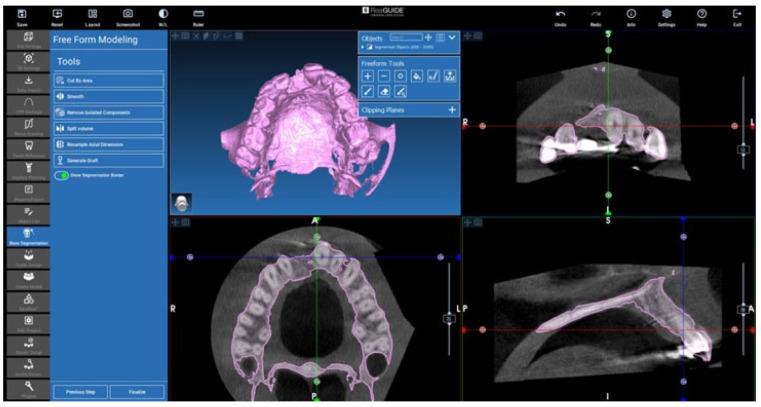
The 3D voxel file in STL format, free form modelling.

Segmentation is a procedure that allows for turning a DICOM image in a 3D voxels volume. In these cases, RealGUIDE 5.2 (3DIEMME, Figino Serenza CO, Italy) software and its tools were used to perform the 3D volume extraction. The method is based on a threshold approach: the software is able to identify and select pixels according to their Hounsfield level (Figure 8). Then, it combines the information to obtain a 3D voxel file in STL format. Free form modelling tools are also available in the software for refining the resulting threshold-segmented 3D file (Figure 9). Bone STL files of the area of interest were created for each patient’s pre-surgery and post-surgery CT exams.

In order to obtain information about the volume with and without the xenograft (bone lamina), two different 3D files from the post-surgery CBCT data set were generated:

The 3D volume with the lamina (including xenograft): “**graft volume**”

The 3D volume without the lamina (excluding xenograft): “**no graft volume**”.

STL files superimposition

To compare the 3D files, they were moved to the same reference system. In this case, the pre-surgery file was moved to the post-surgery CBCT exam reference system.

**Figure 10:**
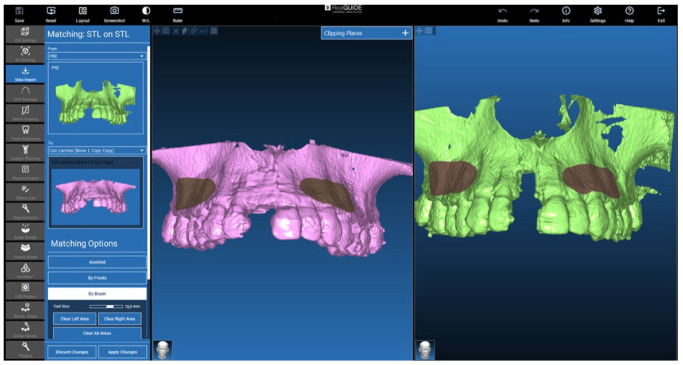
STL on STL matching to move the 3D pre-surgery STL to the post-surgery CT reference system.

The movement was carried out in RealGUIDE 5.2 (3DIEMME, Italy) software, selecting the file surfaces unaffected by the surgery (grey areas in Figure 10). These areas were used to superimpose the 2 STL-files. Starting from these areas in common, the software provided the best matching for the 2 objects. The larger the areas, the better the results the software can provide.

### 2.4. Isolation of the Augmented Area

**Figure 11:**
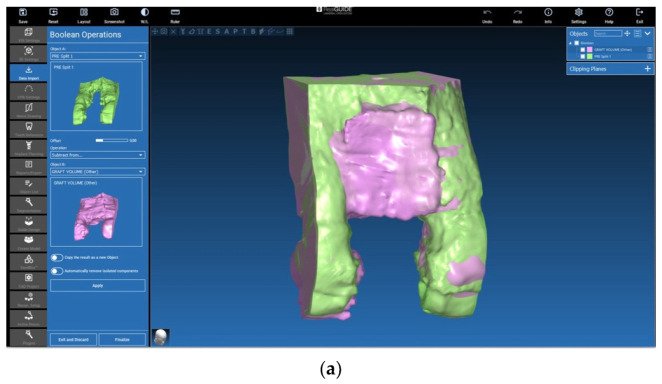

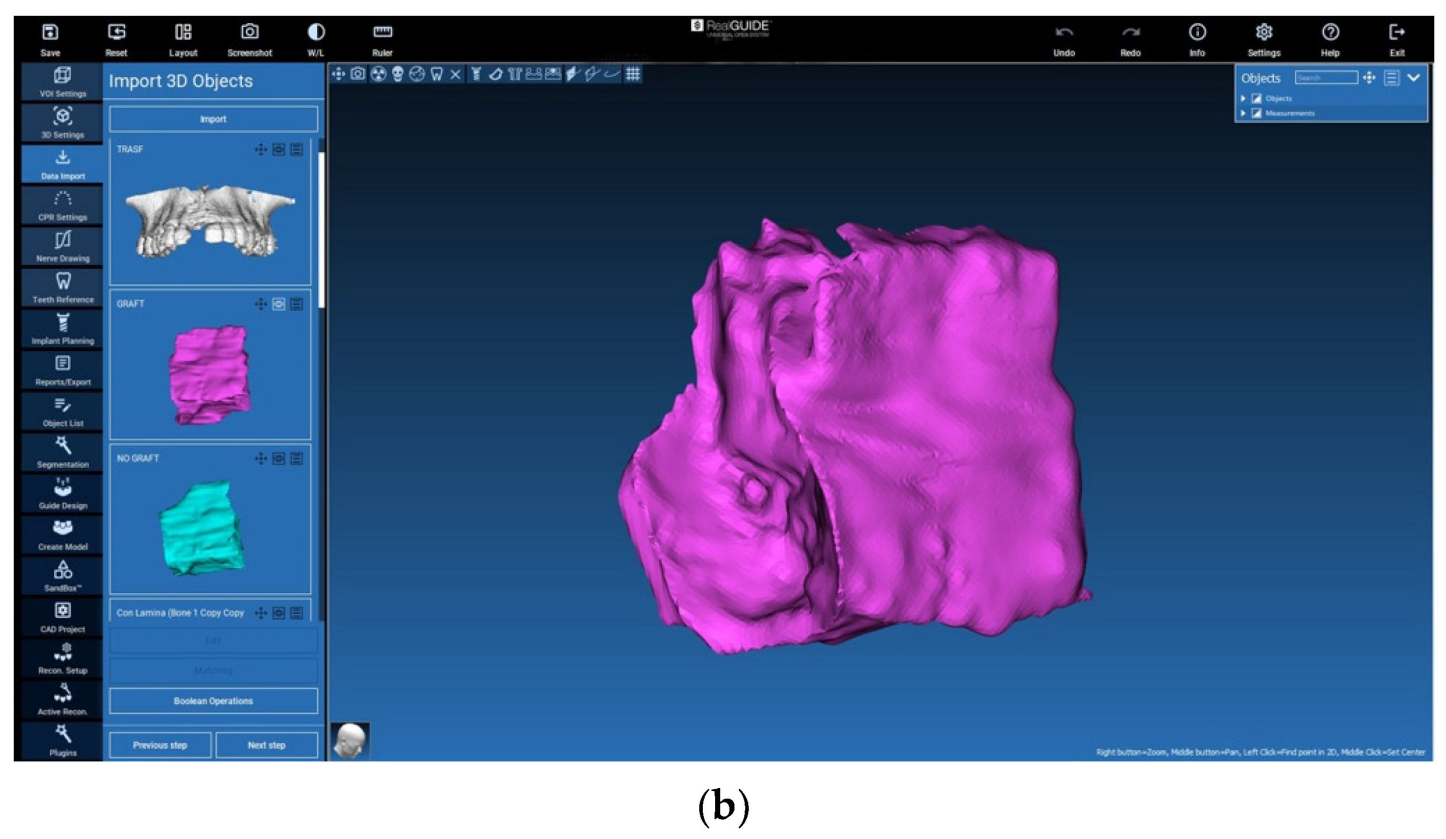
(**a**) Section of interest and Boolean subtraction for isolation of the augmented volume including the lamina (graft). (**b**) Isolated graft portion generation including the lamina (graft).

**Figure 12:**
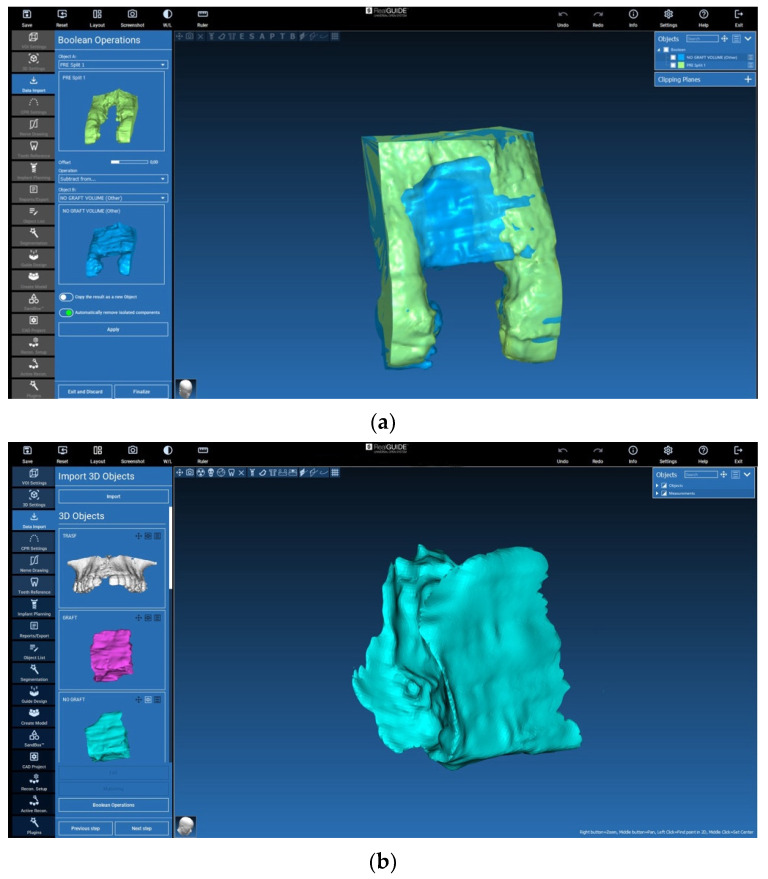
(**a**) Section of interest and Boolean subtraction for isolation of augmented volume without lamina (no graft). (**b**) Boolean subtraction and no graft portion generation.

The 3D files were reduced to a section of interest that included parts of the adjacent structures to the augmented region, e.g., the neighbouring teeth (Figure 11a,b and Figure 12a,b). Then, they were isolated by means of a Boolean subtraction using the pre-surgery STL file. In particular, the following operations were performed:

Graft volume−Pre−surgery volume=Graft portion (Figure 11a,b)
No graft volume−Pre−surgery volume=No graft portion (Figure 12a,b)

This procedure allowed for obtaining the isolated augmented volumes, with (*graft*) and without (*no graft*) the xenograft (lamina).

### 2.5. Evaluation of the Volumes

For each patient, the volume of augmented area was calculated using the tools available in the RealGUIDE 5.2 (3DIEMME, Italy) software. In addition, the difference in volume between the two situations with (“graft volume”) and without (“no graft volume”) the xenograft lamina was calculated. 

**Figure 13:**
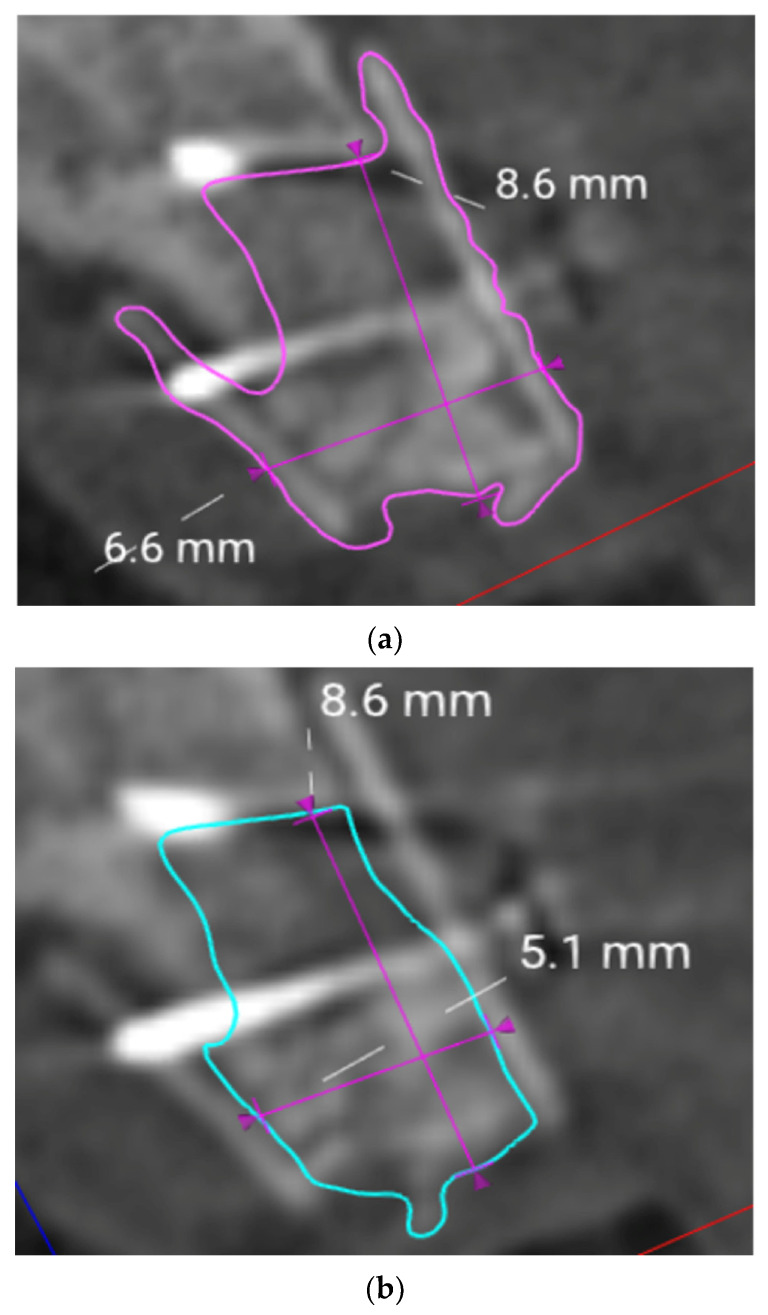
(**a**) Linear measurements within the graft portion. (**b**) Linear measurements within the no graft portion.

Within the designated volumes, linear measurements of the maximum vertical height were obtained (Figure 13a,b).

Descriptive statistical analysis including calculation of the means and standard deviations were performed using IBM SPSS 25 for windows (IBM, Armonk, NY, USA).

## 3. Results

The initial defect size prior to the surgery varied between 7 to 12 mm vertically and 6 to 8 mm horizontally. The healing after all six bone augmentation surgeries proceeded without complication. The augmentation procedures provided sufficient bone for implant installation in the planned position. All of the implants healed in and were restored with an all-ceramic single crown.

The evaluation of the pre- and post-op CBCT scans showed that all the sites improved in bone height and width (Table 1). The mean augmented volume was 382.59 mm^3^ (SD 73.39). The mean augmented volume excluding the lamina was 250.84 mm^3^ (SD 53.67). 

The maximum vertical gain was seen in patient 4 and amounted to 11.3 mm. The mean vertical gain was 8.97 mm (Table 2). 

## 4. Discussion

This case series investigated a novel approach for vertical bone augmentation using a non-invasive digital approach. For this purpose, pre- and post-operative cone beam CT scans from clinical routine were evaluated using special software for surface matching and Boolean subtraction.

Despite the fact that all sites presented Class 5 defects, the surgical approach led to sufficient bone volume that allowed for implant placement and restoration with a single crown, and none of the sites showed complications like infection or excessive resorption. The vertical gain measured in this case series varied from 7 to 11.3 mm. A systematic review and meta-analysis published in 2019 reported on the mean weighted bone gain and complications described for different techniques. Traditional techniques of bone blocks and guided bone regeneration (GBR) showed a relatively minimal mean weighted gain of 3.46 mm (blocks) and 4.18 mm (GBR) with a complication rate of 23.9% and 12.1%, respectively. The complex technique of distraction osteogenesis achieved a relatively high weighted mean of 8.04 mm, but also a high complication rate of 47.3% [2]. 

Hence, the technique applied and assessed in this case series showed its potential to augment a considerable mean bone height of 8.97 mm, while no complications were recorded in this case series. 

Regarding the measured volumes, the results were not easily comparable with other publications. Only a few studies [20,21,22] reported on the amount of autogenous bone harvested and transplanted to a site. Misch et al. [21] published data of a clinical study on bone augmentation and reported a mean volume of 900 mm^3^ (0.9 cm^3^) harvested from the mandibular ramus for onlay bone block grafting. There was no information on the sites that were augmented because the study focused on donor sites. In contrast, von Arx and coworkers [20] reported on bone augmentation limited to single tooth gaps in the maxilla with bone blocks from the ramus. They reported on a mean graft volume of 900 mm^3^ that was obtained from the ramus.

The grafts used in these studies were block grafts. Block grafts undergo a high amount of resorption, which is why sites are always overbuilt in order to compensate for the expected resorption of 20% on average [23,24,25].

The modified shell technique used in this case series allowed for augmenting single tooth sites with severe 3D bone defects using minor bone harvesting. The mean volume that was augmented including the xenogeneic lamina amounted to 382.59 mm^3^ (SD 73.39) and 250.84 mm^3^ (SD 53.67) without the lamina, while a mean vertical augmentation of 8.97 mm was achieved. This shows the effectiveness of the technique. While the original shell technique [26] uses an invasive approach to harvest big bone blocks from the ramus to obtain the shells to reconstruct the buccal and palatal plate, the modified technique limits the harvesting to a bone core, which is taken from the ramus using a trephine. This is because the xenograft lamina is used instead of autogenous bone for the external shells. 

Despite the encouraging results from this case series, limitations have to be discussed. As a result of the small number of only six individuals, the results represent a low level of evidence. The fact that no complications occurred may not reflect clinical routine as patient selection was very strict and the surgeon was very experienced. These results have to be proven in randomized controlled studies with a greater number of individuals, as well as long-term studies. Moreover, biological aspects like the fate of the xenograft lamina and amount of vital bone in the autogenous graft have to be assessed using histo-morphometric methods [8,9]. If the technique proves to be reliable in further research, it could present multiple advantages over traditional techniques. A prerequisite for the regeneration of defects, like the ones treated in this case series, is graft stabilization. This can be achieved with titanium-reinforced membranes, titanium meshes [2], or the traditional SBB technique [10]. Titanium-reinforced membranes and meshes are not resorbable and have to be removed, and they carry the risk of exposure, all of which has been described in the literature [27]. The traditional SBB technique requires extensive bone harvesting in order to obtain bone shells of the required size. As the xenograft lamina is resorbable and available in large numbers, it might facilitate the treatment, limit the extent of bone harvesting, and avoid large surgeries to remove the non-resorbable materials for GBR.

In the past, systematic reviews on bone augmentation used to focus on success rates, complications, and implant survival [28,29]. While different techniques have found their way into the clinical practice, the amount of bone gain that can be achieved has become an important criterion in order to compare different approaches. Today, systematic reviews include an analysis of the linear measurements or percentage of defect fill, but not the augmented total volume [23,30,31,32]. This is because clinical studies usually do not provide these data. However, the amount or volume of the augmented bone may produce a considerable difference regarding the invasiveness and operation time of the procedure. In addition, the nature of the site—single tooth versus two or more missing teeth—will make a difference regarding how demanding an augmentation is. 

This is one reason the technique applied for volumetric evaluation of the augmented sites in this case series is important. Also, it allows for using existing data from the CBCTs that are taken during clinical routine, in order to deliver substantial information on the amount of bone that has been created with a specific technique. By matching the two digital data sets, it allows for precise measuring in one data set and for calculating the obtained volume. These matching procedures have proven to be of high precision [33].

Traditional techniques fail to compare changes in anatomy in one data set. Clinical techniques for the measurement of bone height and width usually involve clinical measurements with calipers or probes [10,34,35,36] at two different time points and encounter the problem of defining reference points. 

In this case series, no CBCT scans directly after surgery were available. Only scans taken after several months of healing were available. Therefore, we cannot provide any information on the amount resorption that took place during the healing process. CBCT scans are usually not taken directly after surgery, in order to avoid unnecessary radiation for the patient. It may be interesting for further study designs to include other non-invasive techniques to collect information on the amount of graft resorption.

The digital technique used in this case series allowed for measuring linear distances in a before-and-after manner in one data set. This was achieved by superimposing the digital 3D data. Only a few authors have reported on comparable digital matching approaches to evaluate bone grafting procedures [37,38,39]. Surface matching and Boolean subtraction, as performed in this study, are known to be a reliable technique of high precision in craniofacial surgical planning [18,40]. Hence, future clinical studies could profit from this technique as it could provide additional information and more accurate data.

## 5. Conclusions

The modified shell technique used in this case series for the vertical augmentation of single-tooth class 5 defects provided sufficient bone for single implant restorations. To evaluate the regenerated new ridge, a digital approach that matched and superimposed digital data from CBCTs taken during the clinical routine was used. This method allowed for linear measurements and calculation of the volume of augmented bone in one data set.

## Figures and Tables

**Table 1:** Results of the volumetric analysis.

Patient No.	Total Augmented Volume (Graft Volume) (mm^3^)	Augmented Volume without Lamina (No Graft Volume) (mm^3^)
1	310.26	219.12
2	405.06	246.24
3	301.68	173.68
4	460.53	334.14
5	468.91	262.15
6	349.09	269.72
Mean	382.59	250.84
Standard Deviation	73.39	53.67

**Table 2:** Results of the linear measurements for vertical gain.

Patient No.	Maximum Vertical Gain (mm)
1	7.4
2	8.6
3	7
4	11.3
5	8.9
6	10.6
Mean	8.97
Standard Deviation	1.71

## Data Availability

The data that support the findings of this study are available from the corresponding author upon reasonable request.

## List of Abbreviations

ASA	American Society of Anesthesiologists
CBCT	cone beam computed tomography
DICOM	digital imaging and communications in medicine
GBR	guided bone regeneration
SBB	split bone block technique
SD	standard deviation
STL	standard tessellation language

## References

[1] Fontana F., Maschera E., Rocchietta I., Simion M. (2011). Clinical classification of complications in guided bone regeneration procedures by means of a nonresorbable membrane. Int. J. Periodontics Restor. Dent..

[2] Urban I.A., Montero E., Monje A., Sanz-Sanchez I. (2019). Effectiveness of vertical ridge augmentation interventions: A systematic review and meta-analysis. J. Clin. Periodontol..

[3] Wang H.L., Boyapati L. (2006). “Pass” principles for predictable bone regeneration. Implant. Dent..

[4] Wikesjo U.M., Kean C.J., Zimmerman G.J. (1994). Periodontal repair in dogs: Supraalveolar defect models for evaluation of safety and efficacy of periodontal reconstructive therapy. J. Periodontol..

[5] Urban I.A., Monje A., Lozada J., Wang H.L. (2017). Principles for vertical ridge augmentation in the atrophic posterior mandible: A technical review. Int. J. Periodontics Restor. Dent..

[6] Benic G.I., Hammerle C.H. (2014). Horizontal bone augmentation by means of guided bone regeneration. Periodontol. 2000.

[7] Fekry Y.E., Mahmoud N.R. (2023). Vertical ridge augmentation of atrophic posterior mandible with corticocancellous onlay symphysis graft versus sandwich technique: Clinical and radiographic analysis. Odontology.

[8] Spin-Neto R., Stavropoulos A., Coletti F.L., Faeda R.S., Pereira L.A., Marcantonio E. (2014). Graft incorporation and implant osseointegration following the use of autologous and fresh-frozen allogeneic block bone grafts for lateral ridge augmentation. Clin. Oral Implant. Res..

[9] Rocchietta I., Simion M., Hoffmann M., Trisciuoglio D., Benigni M., Dahlin C. (2016). Vertical bone augmentation with an autogenous block or particles in combination with guided bone regeneration: A clinical and histological preliminary study in humans. Clin. Implant. Dent. Relat. Res..

[10] Khoury F., Hanser T. (2019). Three-dimensional vertical alveolar ridge augmentation in the posterior maxilla: A 10-year clinical study. Int. J. Oral Maxillofac. Implant..

[11] Urban I.A., Lozada J.L., Wessing B., Suarez-Lopez del Amo F., Wang H.L. (2016). Vertical bone grafting and periosteal vertical mattress suture for the fixation of resorbable membranes and stabilization of particulate grafts in horizontal guided bone regeneration to achieve more predictable results: A technical report. Int. J. Periodontics Restor. Dent..

[12] Khoury F., Khoury C., Khoury F., Antoun H., Missika P. (2007). Mandibular bone block grafts: Diagnosis instrumentation, harvesting techniques and surgical procedures. Bone Augmentation in Oral Implantology.

[13] Rinna C., Ungari C., Saltarel A., Cassoni A., Reale G. (2005). Orbital floor restoration. J. Craniofac. Surg..

[14] Ozel B., Findikcioglu K., Sezgin B., Guney K., Barut I., Ozmen S. (2015). A new option for the reconstruction of orbital floor defects with heterologous cortical bone. J. Craniomaxillofac. Surg..

[15] Rossi R., Rancitelli D., Poli P.P., Rasia Dal Polo M., Nannmark U., Maiorana C. (2016). The use of a collagenated porcine cortical lamina in the reconstruction of alveolar ridge defects. A clinical and histological study. Minerva Stomatol..

[16] Wismeijer D., Joda T., Flügge T., Fokas G., Tahmaseb A., Bechelli D., Bohner L., Bornstein M., Burgoyne A., Caram S. (2018). Group 5 iti consensus report: Digital technologies. Clin. Oral Implant. Res..

[17] Gallucci G.O., Evans C., Tahmaseb A., Wismeijer D., Barter S., Donos N. (2019). Iti Treatment Guide; Digital Workflows in Implant Dentistry.

[18] Charton J., Laurentjoye M., Kim Y. (2017). 3D boolean operations in virtual surgical planning. Int. J. Comput. Assist. Radiol. Surg..

[19] Mak P.H., Campbell R.C., Irwin M.G. (2002). The asa physical status classification: Inter-observer consistency. American society of anesthesiologists. Anaesth. Intensive Care.

[20] von Arx T., Buser D. (2006). Horizontal ridge augmentation using autogenous block grafts and the guided bone regeneration technique with collagen membranes: A clinical study with 42 patients. Clin. Oral Implant. Res..

[21] Misch C.M. (1997). Comparison of intraoral donor sites for onlay grafting prior to implant placement. Int. J. Oral Maxillofac. Implant..

[22] Happe A. (2007). Use of a piezoelectric surgical device to harvest bone grafts from the mandibular ramus: Report of 40 cases. Int. J. Periodontics Restor. Dent..

[23] Esposito M., Grusovin M.G., Coulthard P., Worthington H.V. (2006). The efficacy of various bone augmentation procedures for dental implants: A cochrane systematic review of randomized controlled clinical trials. Int. J. Oral Maxillofac. Implant..

[24] Herford A.S., Nguyen K. (2015). Complex bone augmentation in alveolar ridge defects. Oral Maxillofac. Surg. Clin. N. Am..

[25] Bahat O., Fontanessi R.V. (2001). Implant placement in three-dimensional grafts in the anterior jaw. Int. J. Periodontics Restor. Dent..

[26] Khoury F., Antoun H., Missika P. (2007). Bone Augmentation in Oral Implantology.

[27] Cunha G., Carvalho P.H.A., Quirino L.C., Torres L.H.S., Filho V.A.P., Gabrielli M.F.R., Gabrielli M.A.C. (2022). Titanium mesh exposure after bone grafting: Treatment approaches-a systematic review. Craniomaxillofac Trauma. Reconstr..

[28] Chiapasco M., Zaniboni M., Boisco M. (2006). Augmentation procedures for the rehabilitation of deficient edentulous ridges with oral implants. Clin. Oral Implant. Res..

[29] Aghaloo T.L., Moy P.K. (2007). Which hard tissue augmentation techniques are the most successful in furnishing bony support for implant placement?. Int. J. Oral Maxillofac. Implant..

[30] Naenni N., Lim H.C., Papageorgiou S.N., Hammerle C.H.F. (2019). Efficacy of lateral bone augmentation prior to implant placement: A systematic review and meta-analysis. J. Clin. Periodontol..

[31] Urban I.A., Saleh M.H.A., Ravida A., Forster A., Wang H.L., Barath Z. (2021). Vertical bone augmentation utilizing a titanium-reinforced ptfe mesh: A multi-variate analysis of influencing factors. Clin. Oral Implant. Res..

[32] Troeltzsch M., Troeltzsch M., Kauffmann P., Gruber R., Brockmeyer P., Moser N., Rau A., Schliephake H. (2016). Clinical efficacy of grafting materials in alveolar ridge augmentation: A systematic review. J. Craniomaxillofac. Surg..

[33] Ritter L., Reiz S.D., Rothamel D., Dreiseidler T., Karapetian V., Scheer M., Zoller J.E. (2012). Registration accuracy of three-dimensional surface and cone beam computed tomography data for virtual implant planning. Clin. Oral Implant. Res..

[34] Maiorana C., Beretta M., Salina S., Santoro F. (2005). Reduction of autogenous bone graft resorption by means of bio-oss coverage: A prospective study. Int. J. Periodontics Restor. Dent..

[35] Wachtel H., Fickl S., Hinze M., Bolz W., Thalmair T. (2013). The bone lamina technique: A novel approach for lateral ridge augmentation—A case series. Int. J. Periodontics Restor. Dent..

[36] Merli M., Migani M., Esposito M. (2007). Vertical ridge augmentation with autogenous bone grafts: Resorbable barriers supported by ostheosynthesis plates versus titanium-reinforced barriers. A preliminary report of a blinded, randomized controlled clinical trial. Int. J. Oral Maxillofac. Implant..

[37] Velázquez Ó.I., Tresguerres F.G.F., Berrocal I.L., Tresguerres I.F., López-Pintor R.M., Carballido J., López-Quiles J., Torres J. (2021). Split bone block technique: 4-month results of a randomised clinical trial comparing clinical and radiographic outcomes between autogenous and xenogeneic cortical plates. Int. J. Oral Implant..

[38] Merli M., Moscatelli M., Mazzoni A., Mazzoni S., Pagliaro U., Breschi L., Motroni A., Nieri M. (2013). Fence technique: Guided bone regeneration for extensive three-dimensional augmentation. Int. J. Periodontics Restor. Dent..

[39] Schnutenhaus S., Martin T., Dreyhaupt J., Rudolph H., Luthardt R.G. (2018). Dimensional changes of the soft tissue after alveolar ridge preservation with a collagen material. A clinical randomized trial. Open Dent. J..

[40] Vannier M.W. (2000). Evaluation of 3D imaging. Crit. Rev. Diagn. Imaging.

